# Neurologic complications of sickle cell disease in Africa: A systematic review and meta-analysis

**DOI:** 10.1212/WNL.0000000000004537

**Published:** 2017-10-03

**Authors:** Jean Jacques Noubiap, Michel K. Mengnjo, Nicolas Nicastro, Joseph Kamtchum-Tatuene

**Affiliations:** From the Department of Medicine (J.J.N.), Groote Schuur Hospital and University of Cape Town, South Africa; Department of Medicine and Medical Specialties, Faculty of Medicine and Biomedical Sciences (M.K.M.), University of Yaoundé, Cameroon; Division of Neurorehabilitation, Department of Clinical Neurosciences (N.N.), Geneva University Hospitals, Switzerland; Brain Infections Group, Institute of Infection and Global Health (J.K.-T.), University of Liverpool; and Department of Neurology (J.K.-T.), The Walton Centre for Neurology and Neurosurgery, Liverpool, UK.

## Abstract

**Objective:**

To summarize prevalence data on the neurologic complications of sickle cell disease (SCD) in Africa.

**Methods:**

We searched EMBASE, PubMed, and African Index Medicus to identify all relevant articles published from inception to May 31, 2016. Each study was reviewed for methodologic quality. A random-effects model was used to estimate the prevalence of neurologic complications of SCD across studies.

**Results:**

Thirty-one studies were included. Methodologic quality was high or moderate in 90% of studies. Stroke, conditional and abnormal cerebral blood flow, seizures, and headache were the complications most frequently reported, with overall prevalence rates of 4.2%, 10.6%, 6.1%, 4.4%, and 18.9%, respectively. Some complications, like silent brain infarcts, peripheral neuropathies, neurocognitive deficits, or moyamoya disease, have been rarely or not studied at all in the African setting. Incidence data were scarce and of poor quality.

**Conclusions:**

The burden of neurologic complications of SCD is important in Africa and most likely underestimated. A better evaluation of this burden requires larger prospective studies using standard up-to-date screening methods. Accessibility to diagnostic tools such as neuroimaging, transcranial Doppler, EEG, and neuropsychological evaluation, as well as to preventive and therapeutic interventions and trained health care providers, should be improved in routine clinical practice.

Sickle cell disease (SCD) affects 83% of the 330,000 babies born each year with a major hemoglobinopathy worldwide. Of the 20–25 million people living with SCD worldwide, 12–15 million live in Africa.^1,2^

By decreasing order of frequency, neurologic complications of SCD include silent brain infarcts (39% by age 18), acute and chronic headache (36% in children), neurocognitive impairment (25%), seizures (7%–10%), ischemic stroke (1% in children with effective screening and prophylaxis, but nearly 11% in children without screening), and hemorrhagic stroke (3% in children and 10% in adults).^3–5^ These data are mainly derived from populations residing outside Africa and little has been done to provide a more accurate evaluation of the burden of SCD and its complications in Africa, which has the highest prevalence of the disease, despite the resolution adopted by the 63rd session of the UN General Assembly recognizing SCD as a major public health concern.^6^

Therefore, we conducted the first systematic review and meta-analysis to summarize prevalence data on the neurologic complications of SCD in Africa and to identify knowledge gaps that may be addressed in future studies.

## METHODS

The Preferred Reporting Items for Systematic Reviews and Meta-Analyses guidelines served as the template for reporting the review. This review was registered in the PROSPERO international prospective register of systematic reviews (registration number CRD42016039574) and its protocol was published.^7^

### Literature search.

We performed a search of PubMed/MEDLINE, EMBASE, and African Index Medicus to identify all relevant articles reporting data on the neurologic complications of SCD in Africa published from inception to May 31, 2016. No language restrictions were applied. Details of the search strategy have been reported previously.^7^

### Selection of studies for inclusion in the review.

We included all observational studies and clinical trials reporting the prevalence and incidence of the neurologic complications of SCD in an African population. Studies conducted on non-African populations, small case series (<30 participants), letters, reviews, and editorials were excluded. Details of the study selection process have been reported previously.^7^

**Figure 1:**
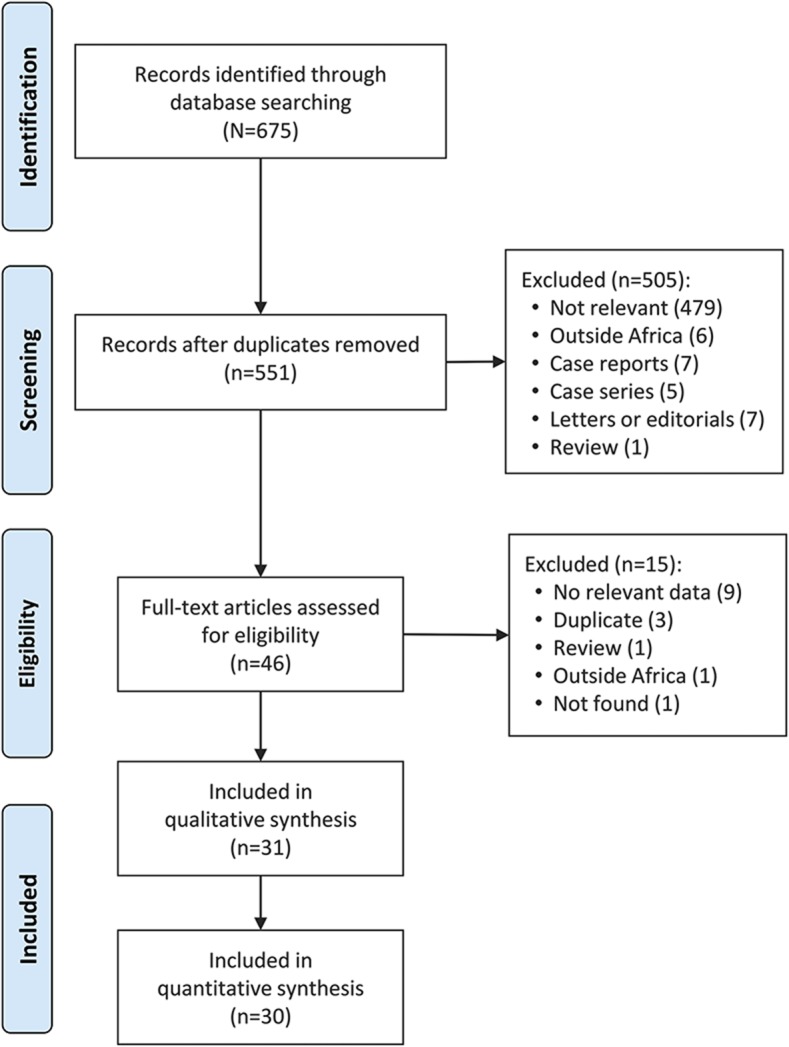
Flowchart summarizing the article selection process

Two investigators (J.J.N., J.K.-T.) independently screened the titles and abstracts of the articles identified during the initial literature search, and then the full text of articles found potentially eligible were obtained and further assessed for final inclusion (figure 1). Disagreements were resolved through consensus following a discussion between the 2 assessors. The interrater agreement for the selection of studies was assessed using a nonweighted Cohen kappa. The Cohen kappa statistic is a measure of the true agreement between 2 observers. It indicates the proportion of the possible beyond chance agreement that is attributable to the individual performance of the observers.^8–10^

### Appraisal of the risk of bias.

Two investigators (J.J.N., J.K.-T.) independently assessed the methodologic quality and the risk of bias for each study by using a 10-point rating system as described previously.^7,11^ Studies were rated as having a low, intermediate, or high risk of bias when they were assigned a score higher than 8, between 6 and 8, or lower than 6, respectively.^7,11^ Disagreements on the category of bias were resolved through consensus following a discussion between the 2 assessors. The interrater agreement for the score and the category of bias assigned was assessed using a weighted Cohen kappa. A linear weighting system was used in this case. When the decision of the observers is expressed as an ordinal categorical variable with at least 3 possible values, a weighted Cohen kappa statistic is preferred because it accounts for the magnitude of the disagreement.^12,13^

### Data extraction and management.

For each study, the following information was collected: surname of the first author, year of publication, PubMed accession number (pmid), country and region of Africa where the study was conducted, study setting (urban vs rural, hospital-based vs community-based), study design, duration of follow-up for cohort studies, sample size, mean age and age range, proportion of male participants, diagnostic test for SCD, operational definition, diagnostic method, and prevalence of each complication reported. Data were extracted using a predesigned form (appendix e-1 at Neurology.org). At the end of the extraction process, 2 investigators (J.J.N., J.K.-T.) checked all the data for accuracy. Disagreements were solved through consensus following a discussion between the 2 assessors.

### Statistical analyses.

The meta-analyses were carried out with the software STATA (version 13; StataCorp, College Station, TX) using the metaprop command.^14^ The heterogeneity between studies was evaluated with the Cochran Q test and quantified using the *I*^*2*^ index.^15^ *I*^*2*^ values of 25%, 50%, and 75% were interpreted as low, medium, and high heterogeneity, respectively. The confidence intervals for the study-specific prevalence were computed using the score method.^14,16^ Due to the high heterogeneity between studies, pooled prevalence of the various neurologic complications of SCD were computed using a random effect model^17^ after stabilizing the variance with the Freeman-Tukey double arcsine transformation.^14,18,19^ Results of the meta-analyses were presented as forest plots. Sensitivity analyses were performed to evaluate the influence of each study or subgroup of studies on overall prevalence estimates and to identify potential sources of heterogeneity. The subgroups were predefined in the protocol.^7^ Assessment of the small-study effects was carried out by combining visual inspection of funnel plots with the Egger test.^20^ Statistical significance was defined as *p* < 0.05.

## RESULTS

### Study selection.

The initial literature search retrieved 675 articles, of which 46 were selected for full-text review. Of these, 31 were eligible and included in this systematic review (figure 1).^21–51^ There was 93.8% agreement between investigators for study inclusion (κ = 0.55).

### Characteristics of included studies.

All the studies included were hospital-based and conducted in 14 countries distributed across 4 of the 5 WHO subregions of Africa with reports published between 1977 and 2016 (table e-1). Ninety percent of the studies were classified as having a low or moderate risk of bias (table e-1). There was 93.6% agreement between investigators for the scoring of methodologic quality and risk of bias (κ = 0.75).

### Pooled prevalence and incidence of the complications most frequently reported.

#### Stroke.

**Figure 2:**
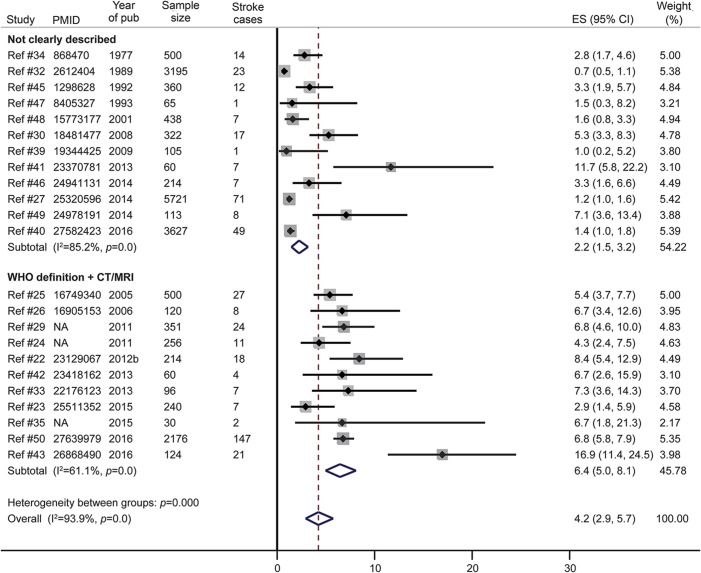
Meta-analysis of the prevalence of stroke in sickle cell disease according to the diagnostic criteria CI = confidence interval; ES = effect size; NA = not available (article not indexed in PubMed/no accession number available).

The overall prevalence of stroke in SCD was 4.2% in a pooled sample of 18,977 participants from 23 studies (figure 2). This prevalence was higher in studies with smaller sample size (figure e-1) and the Egger test for small study effects was significant (bias statistic = 0.64, *p* = 0.005, funnel plot provided as figure e-2). The prevalence of stroke in SCD was also higher in the 11 studies using more reliable diagnostic criteria for stroke (WHO clinical case definition or brain CT or MRI), representing a pooled sample of 4,257 patients with SCD as shown in figure 2. Finally, the prevalence of stroke in SCD was higher in studies published after 2011 (figure e-3). Only one study investigated the incidence rate of stroke in SCD. This was 3 per 100 person-years in a sample of 66 patients aged 37–197 months and followed up over 12 months.^21^

The type of stroke (ischemic vs hemorrhagic) was reported in 4 studies.^22–25^ In one of these studies, a brain CT was performed in all patients with stroke, revealing an ischemic lesion in 94.4% (17/18) of cases.^22^ In the other 3 studies, brain CT/MRI was performed only for a few patients with clinical features of stroke (4/5, 3/11, and 7/25 patients, respectively)^23–25^; the stroke was ischemic in all cases with brain imaging (4/4 and 3/3) in 2 studies,^23,24^ and in 71.4% (5/7) of cases in the third.^25^

The 5 studies reporting data on stroke recurrence are listed in table e-2.^22,24,26–28^ The prevalence of recurrent stroke varied from 18.2% (2/11) to 77.8% (14/18). In one study,^28^ the incidence of stroke recurrence was 28/100 person-years in 18 patients not taking hydroxyurea and 7/100 person-years in 13 patients on hydroxyurea. Children who did not receive hydroxyurea were more likely to drop out of school and to have moderate to severe motor disabilities requiring caregiver assistance for daily living. There was no difference in the rate of children developing epilepsy during the 30-month follow-up (23.1% in children on hydroxyurea vs 16.7% in children not taking hydroxyurea).

Two other studies reported on long-term sequelae of stroke in children with SCD.^25,29^ Speech impairment (aphasia or dysphasia) was found in 12% (3/25) and 73.9% (17/23), intellectual disability in 8% (2/25) and 52.2% (12/23), motor disability (hemiparesis or quadriparesis) in 76% (19/25) and 83.3% (20/23), and seizures/epilepsy in 24% (6/25) and 30.4% (7/23) of children who survived after a stroke.

#### Conditional or abnormal cerebral blood flow.

**Figure 3:**
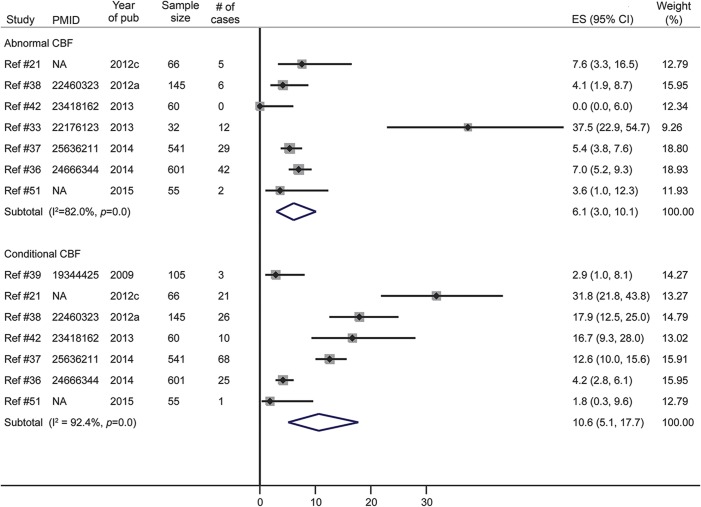
Meta-analysis of the prevalence of conditional/abnormal cerebral blood flow (CBF) in sickle cell disease CI = confidence interval; ES = effect size; NA = not available (article not indexed in PubMed/no accession number available).

Cerebral blood flow (CBF) in people with SCD was investigated in 7 studies using transcranial Doppler to obtain the time-averaged mean of the maximum velocities (TAMM). The overall prevalence of conditional CBF (TAMM between 170 and 200 cm/s) was 10.6% in a pooled sample of 1,573 participants from 6 studies (figure 3). The overall prevalence of abnormal CBF (TAMM >200 cm/s) was 6.1% in a pooled sample of 1,500 participants from 7 studies (figure 3). The Egger test for small-study effect was not significant in either subgroup (bias statistic = 0.87 and 0.45, respectively; *p* = 0.40 and 0.50, respectively).

#### Seizures.

**Figure 4:**
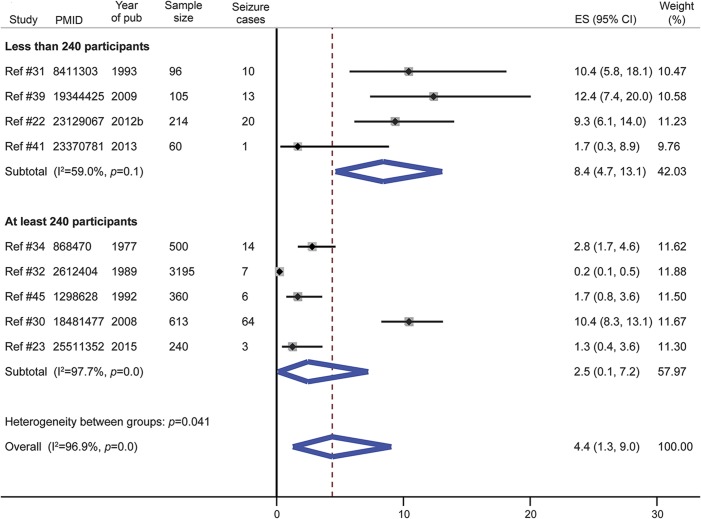
Meta-analysis of the prevalence of seizure in sickle cell disease according to the study size CI = confidence interval; ES = effect size.

The prevalence of seizures was reported in 9 studies, with 4 making a clear description of the etiology (febrile convulsions, provoked seizures, single symptomatic seizures, epilepsy)^22,30–32^ and only 2 using EEG.^22,31^ The overall prevalence of seizures in SCD was 4.4% in a pooled sample of 5,383 patients (figure 4). This prevalence was higher in studies with smaller sample size and there was significant intergroup heterogeneity (heterogeneity statistic = 4.2, *p* = 0.04). The Egger test for small study effect was not significant (bias statistic = 1.02, *p* = 0.08). The prevalence of seizures in SCD was lower in studies published before 2008 (figure e-4). Specifically, in a pooled sample of 3,505 patients from 3 studies, the prevalence of febrile convulsions was 3.0% (figure e-5) and that of epilepsy was 2.8% (figure e-6).

#### Other neurologic complications.

**Figure 5:**
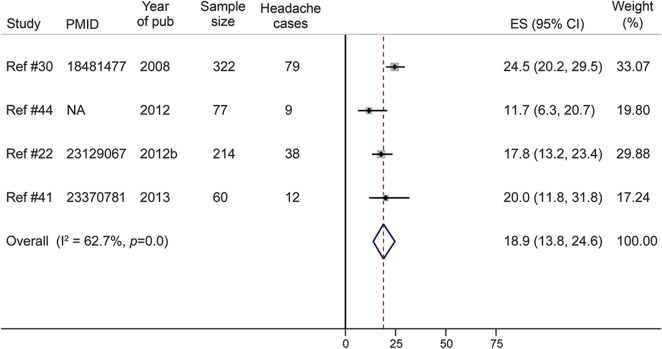
Meta-analysis of the prevalence of headache in patients with sickle cell disease CI = confidence interval; ES = effect size; NA = not available (article not indexed in PubMed/no accession number available).

The prevalence of recurrent headache in SCD was 18.9% in a pooled sample of 673 patients from 4 studies (figure 5). One study in Cameroon evaluated the cognitive function (executive function, attention, memory, and motor skills) of 96 patients with SCD aged 6–24 years (mean 13.5, SD 4.9). Mild or severe cognitive deficits were reported in 15 (15.6%) and 21 (21.9%) patients, respectively.^33^ In another study in the Democratic Republic of Congo, 17.5% (7/40) of patients with SCD were found to have cognitive and motor deficits.^34^

A Nigerian study found a prevalence of localized sensory neuropathy of 2.1% (2/96), 16.8% (38/226), and 4.1% (12/291) among children (4–14 years), adolescents (15–19 years), and adults (≥20 years) with SCD, respectively, giving an overall prevalence of 8.5% (52/613).^30^ One study reported the prevalence of silent brain infarcts, which was 16.7% (5/30) in patients with SCD younger than 18 years.^35^

## DISCUSSION

This systematic review and meta-analysis shows that stroke, conditional and abnormal CBF, seizures, and headache are the neurologic complications of SCD most frequently reported, with an overall prevalence of 4.2%, 10.6%, 6.1%, 4.4%, and 18.9%, respectively. It also highlights the fact that some complications like silent brain infarcts, peripheral neuropathies, neurocognitive deficits, and moyamoya disease have been studied rarely or not at all in the African setting. Overall, the epidemiology of neurologic complications in SCD seems to be different in patients residing in Africa when compared to those not residing in Africa.

The prevalence of stroke in SCD is about 3 times higher in studies that used more reliable case definitions and diagnostic tools as compared to those with unclear case definitions (6.4% vs 2.2%). Therefore, the prevalence of stroke in African patients with SCD is probably underestimated, especially when considering the higher prevalence reported in other settings (8%–11%).^5,52^ Consistent with data from other populations,^5^ we found that ischemic lesions were the most frequent type of stroke. Despite being of limited precision (retrospective data collection in small samples), available data show that stroke recurrence is observed in 18%–78% of patients with SCD, with higher recurrence rates recorded in those not taking hydroxyurea,^28^ thus highlighting the potential benefit of this drug for secondary stroke prevention in low to middle income countries where the current standard treatment—chronic transfusion therapy (CTT)^53^—is most often not affordable. Hydroxyurea seems to bear even more hope for patients with SCD in Africa when considering the results of the TCD With Transfusions Changing to Hydroxyurea (TWiTCH) trial demonstrating the noninferiority of hydroxyurea over chronic blood transfusion for primary stroke prevention in SCD after at least 1 year of CTT.^54^ Increasing the accessibility to early transcranial Doppler (TCD) screening and early initiation of hydroxyurea before the first overt stroke could be far more promising options to reduce the burden of stroke in SCD. However, it is not yet known if the beneficial effects of hydroxyurea would be identical if started immediately after the detection of abnormal TCD velocities in CTT-naive patients.^55^ Hopefully the ongoing randomized clinical trial in Nigeria (NCT02560935) will help to fill this knowledge gap.

The pooled prevalence of conditional and abnormal CBF in this study (10.6% and 6.1%, respectively) are far lower than those found in populations outside Africa. For instance, the Centre Hospitalier Intercommunal de Creteil newborn cohort study in France found a prevalence of abnormal CBF of nearly 30%.^56^ Moreover, only one small-sized study in this review investigated the prevalence of silent brain infarcts, reporting a prevalence of 16.7%, which is lower than the prevalence of 39% by 18 years found in non-African populations.^57^ These lower rates of stroke, silent brain infarcts, and abnormal CBF in African patients with SCD might be explained by the limited access to screening and diagnostic tools (TCD, CT, MRI) as well as the poorer documentation and follow-up due to limited availability of trained health personnel who would be able to identify, report, and manage cases adequately.

Seizures represent another common neurologic complication of SCD. Recurrent seizures (epilepsy) are 2–3 times more frequent in individuals with SCD than in the general population, and are associated with premature death.^4,58^ In the Jamaica cohort study of SCD, approximately 7% of individuals had experienced at least 1 seizure.^58^ The frequency of seizures in this meta-analysis (4.4%) is lower and might be explained by the predominance of older studies conducted before the 21st century. These studies provide nearly 80% of the pooled sample used in the meta-analysis of seizures data. It is likely that the data reported in older studies were of poor quality due to low public awareness, limited availability of trained health personnel, and poor access to adequate diagnostic resources. Indeed, the overall prevalence of seizures in studies conducted after 2008 (6.3%) is closer to that obtained in the Jamaica cohort study.^58^

This review reveals that recurrent headache is a common (18.9%) problem in patients with SCD in Africa, though less frequent than reported in high-income settings. For instance, in a large retrospective cross-sectional study in the United States (n = 872), recurrent headache and migraine were found in 36.1% and 15.1% of children with SCD, respectively.^59^ The pathogenesis of headache in SCD remains unclear, but factors like anemia or stress have been suggested as possible mechanisms. Some studies have shown an association between headache and low hemoglobin level, and others have demonstrated an improvement of headache following blood transfusion.^5^

Of the 2 studies assessing neurocognitive deficits in SCD in Africa, one used a well-structured neuropsychological test battery and found a higher frequency of cognitive deficits (37.5%) than usually reported in western countries.^33^ This study conducted in Cameroon also reported a decline in attention and executive functions in late childhood, thus highlighting the need to integrate a long-term neuropsychological assessment as part of the routine care of children with SCD and to discuss the potential benefits of specific cognitive training programs for selected groups. However, further studies are needed to provide a more detailed description of the neurocognitive functions that are affected in patients with SCD in Africa, so as to inform the design of rehabilitation programs.

The interpretation of our results should take into account a few limitations that could alter the accuracy or the precision of our estimations: (1) the substantial heterogeneity between studies in most of the meta-analyses performed, (2) the retrospective data collection in most of the studies included, and (3) the lack of clear case definitions or reliable diagnostic tools in some studies. Nevertheless, the stringent criteria applied during the selection of studies and the rigorous methodology used for data analysis helped to mitigate the effects of these limitations.

The burden of neurologic complications of SCD such as stroke, seizures, neurocognitive deficits, and headache is important in Africa and most likely underestimated. Indeed, the prevalence of these complications are lower than in non-African populations, contrasting with a higher vulnerability of patients with SCD in Africa due to limited access to adequate preventive and therapeutic interventions. The difference is probably due to the inadequacy of the diagnostic methods used in many African studies. Consequently, there is a need for larger prospective studies investigating all neurologic complications of SCD using standard up-to-date screening methods in order to increase the detection rate. Finally, efforts are needed to increase public awareness of neurologic complications of SCD; accessibility to adequate diagnostic tools (neuroimaging, TCD, EEG, neuropsychological evaluation) and preventive and therapeutic interventions; as well as availability of trained health care providers.

## Supplementary Material

Data Supplement

Accompanying Editorial

## GLOSSARY

CBF: cerebral blood flow
CTT: chronic transfusion therapy
SCD: sickle cell disease
TAMM: time-averaged mean of the maximum
TCD: transcranial Doppler
