# Supplementary material for: Neurologic complications of sickle cell disease in Africa: A systematic review and meta-analysis
Source: Neurology. 2017 Oct 3;89(14):1516–24. doi: 10.1212/WNL.0000000000004537 (PMC5631172; doi:10.1212/WNL.0000000000004537)
Supplement: Data Supplement [file supp_WNL.0000000000004537_e-figures.pdf]

Figure e-1: Meta-analysis of the prevalence of stroke in sickle cell disease according to study size

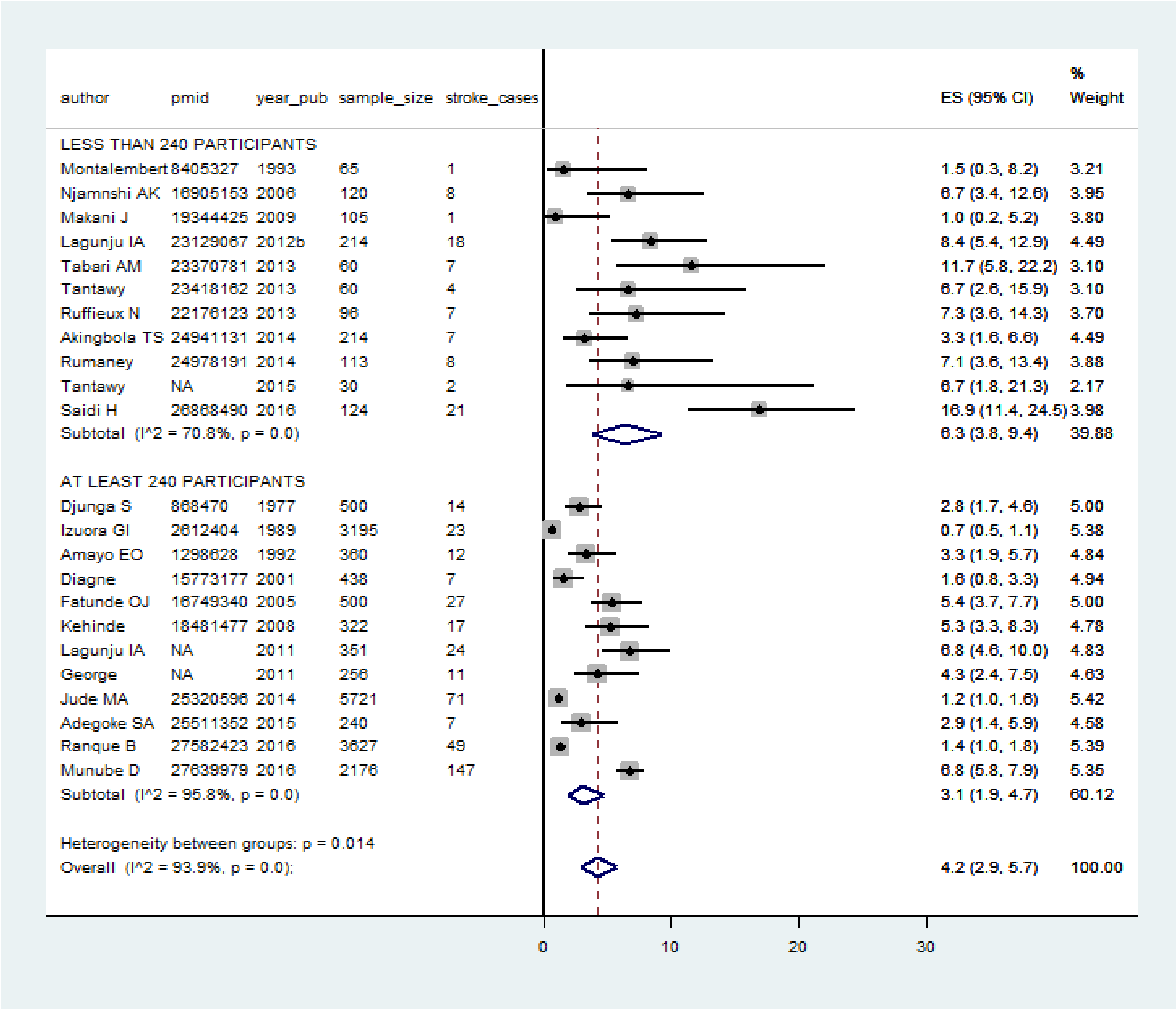

ES = Effect Size, CI = Confidence Interval, NA = Not available (article not indexed in PubMed / No accession number available)

Figure e-2: Funnel plot showing the 23 studies reporting the prevalence of stroke in sickle cell disease

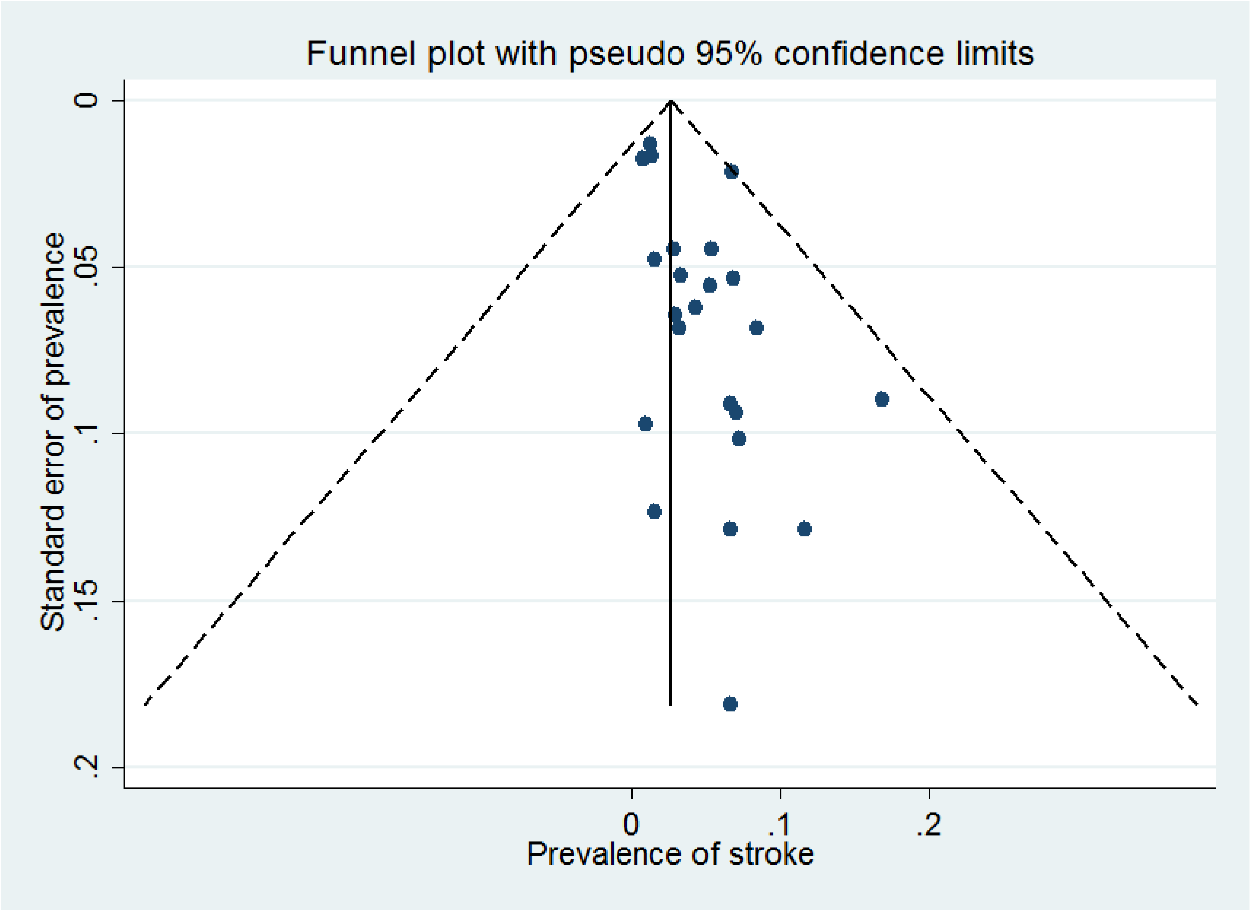

Figure e-3: Meta-analysis of the prevalence of stroke in sickle cell disease according to year of publication

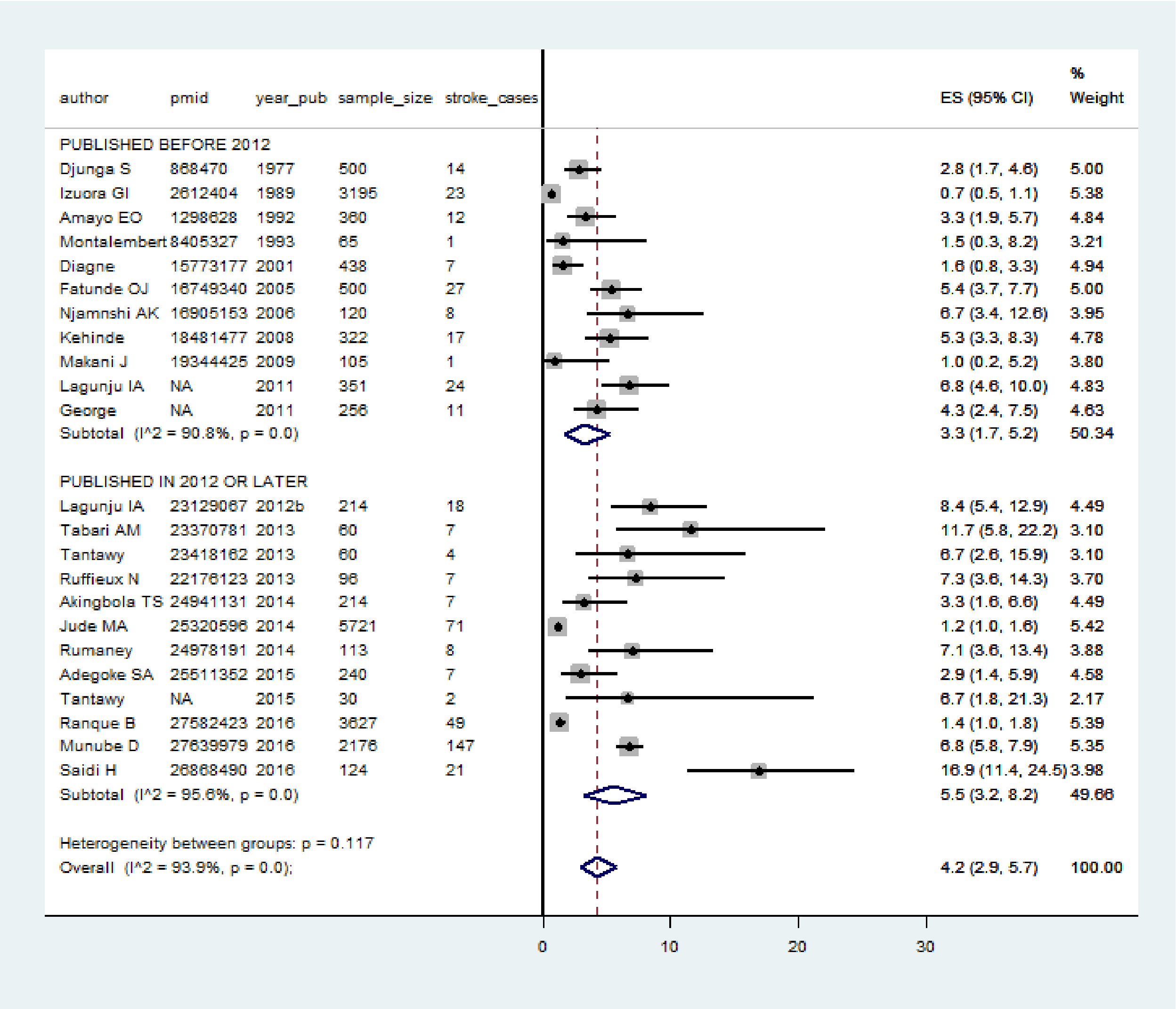

ES = Effect Size, CI = Confidence Interval, NA = Not available (article not indexed in PubMed / No accession number available)

Figure e-4: Meta-analysis of the prevalence of seizure in sickle cell disease according to year of publication

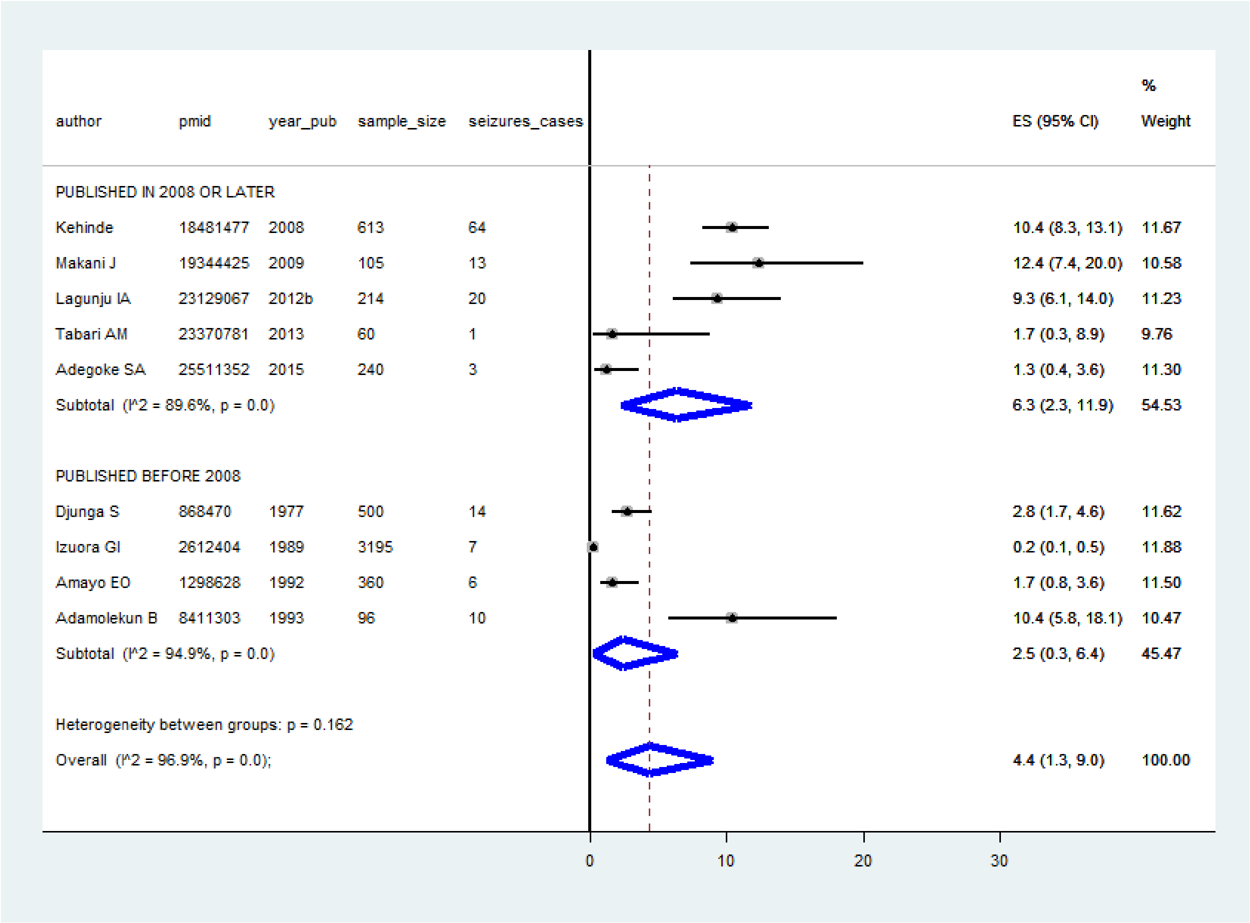

ES = Effect Size, CI = Confidence Interval

Figure e-5: Meta-analysis of the prevalence of febrile convulsions in sickle cell disease

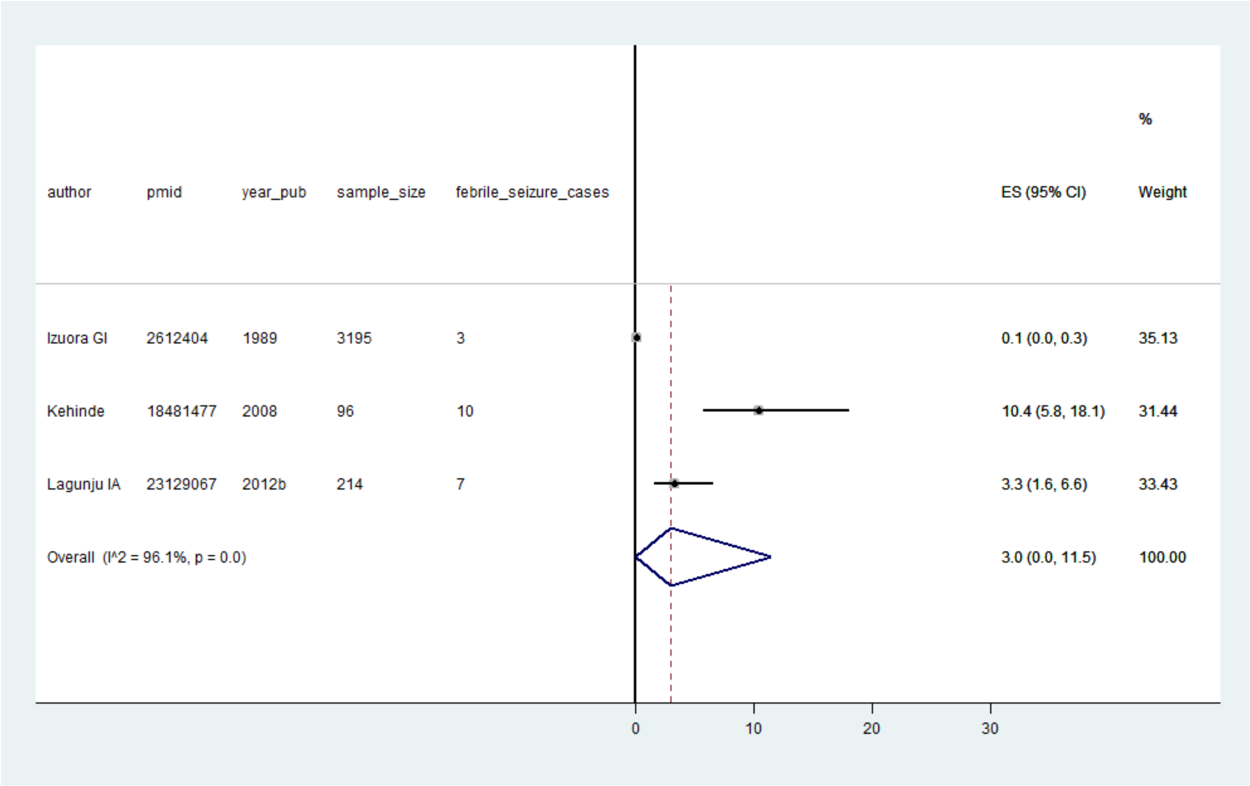

ES = Effect Size, CI = Confidence Interval

Figure e-6: Meta-analysis of the prevalence of epilepsy in sickle cell disease

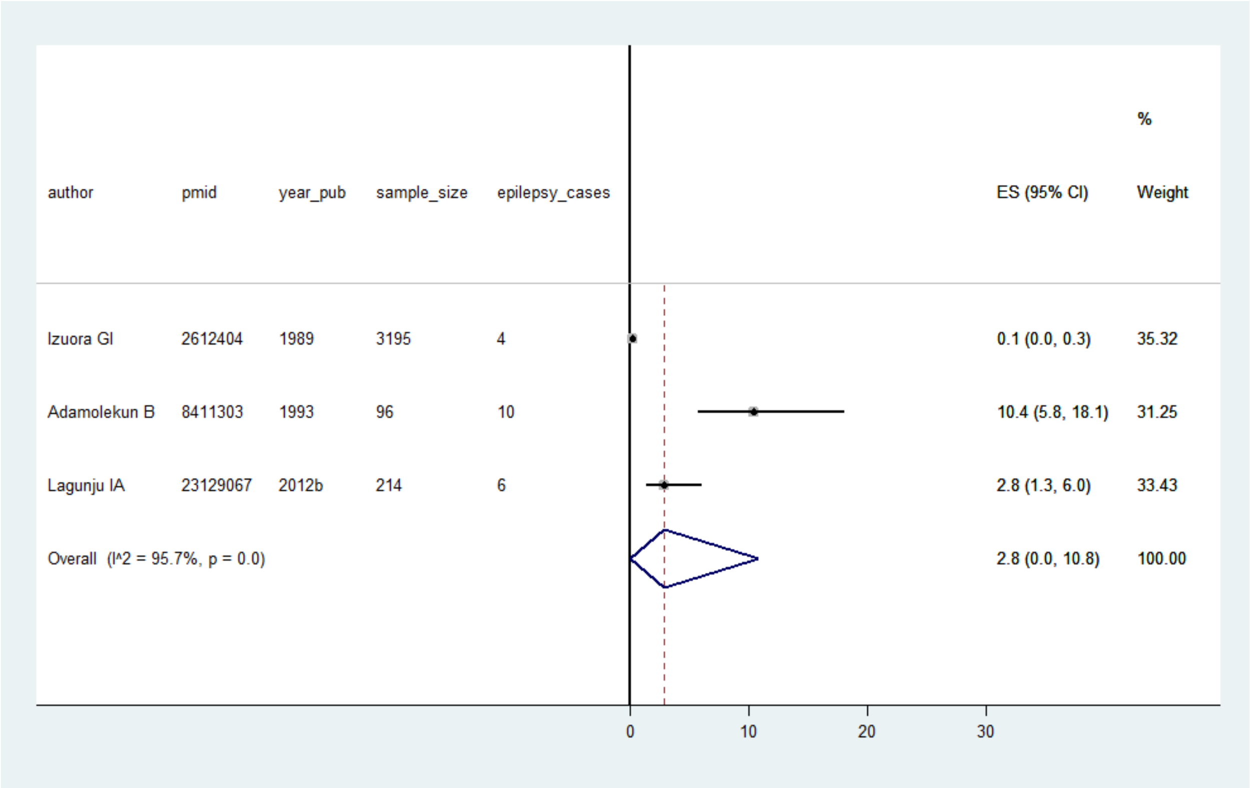

ES = Effect Size, CI = Confidence Interval
