# Supplementary material for: Neurologic complications of sickle cell disease in Africa: A systematic review and meta-analysis
Source: Neurology. 2017 Oct 3;89(14):1516–24. doi: 10.1212/WNL.0000000000004537 (PMC5631172; doi:10.1212/WNL.0000000000004537)
Supplement: Data Supplement [file supp_WNL.0000000000004537_Table_e-1.docx]

**Table e-1: General characteristics of included studies**

| **Author** | **Year of publication** | **Country** | **African sub-region** | **Study design** | **Mode of data collection** | **Random selection** | **Study setting** | **Study area** | **Sex** | **Method of SCD diagnosis** | **Mean age** | **Age range** | **Proportion of males** | **Risk of bias** |
| --- | --- | --- | --- | --- | --- | --- | --- | --- | --- | --- | --- | --- | --- | --- |
| Cox SE | 2014 | Tanzania | Eastern | Cross-sectional | Retrospective | No | Hospital-based | Urban | Both | High performance liquid chromatography (HPLC) | 9.76 | 0,6;22,6 | 54.08 | Low |
| Dorie A | 2014 | Mali | Western | Cross-sectional | Prospective | No | Hospital-based | Urban | Both | High performance liquid chromatography (HPLC) | 7.88 | 1;17 | 56.46 | Low |
| Makani J | 2009 | Kenya | Eastern | Cohort | Prospective | No | Hospital-based | Urban | Both | Hemoglobin electrophoresis | 7.4 | N/A | N/A | Moderate |
| Tabari AM | 2013 | Nigeria | Western | Cross-sectional | Prospective | No | Hospital-based | Urban | Both | Not mentioned | 11.58 | 1;47 | 36.6 | Moderate |
| Lagunju IA_1 | 2012 | Nigeria | Western | Cross-sectional | Prospective | No | Hospital-based | Urban | Both | Hemoglobin electrophoresis | 9.1 | 3;16 | 61.38 | Moderate |
| Lagunju IA_2 | 2012 | Nigeria | Western | Cohort | Prospective | No | Hospital-based | Urban | Both | Hemoglobin electrophoresis | 8.2 | 3;17 | 63.6 | Moderate |
| Lagunju IA_3 | 2012 | Nigeria | Western | Cross-sectional | Prospective | No | Hospital-based | Urban | Both | Hemoglobin electrophoresis | 8.8 | 1;17 | 61.2 | Low |
| Lagunju IA | 2011 | Nigeria | Western | Cross-sectional | Prospective | No | Hospital-based | Urban | Both | Hemoglobin electrophoresis | N/A | N/A | N/A | Low |
| Lagunju IA | 2013 | Nigeria | Western | Cohort | Retrospective | No | Hospital-based | Urban | Both | Hemoglobin electrophoresis | 7.6 | 3;12 | 68.75 | Moderate |
| Tantawy | 2013 | Egypt | Northern | Cross-sectional | Prospective | No | Hospital-based | Urban | Both | High performance liquid chromatography (HPLC) | 10.3 | 3;18 | 56.7 | Moderate |
| Ranque B | 2016 | Cameroon, Gabon, Ivory Coast, Mali, Senegal | Central and western | Cross-sectional | Prospective | No | Hospital-based | Urban | Both | High performance liquid chromatography (HPLC) and Hemoglobin electrophoresis | 16 | >3 yo | 46.4 | Low |
| Elbeshlawy A | 2015 | Egypt | Northern | Cross-sectional | Prospective | No | Hospital-based | Urban | Both | Not mentioned | 10.48 | N/A | N/A | Moderate |
| Munube D | 2016 | Uganda | Eastern | Cross-sectional | Retrospective | No | Hospital-based | Urban | Both | Hemoglobin electrophoresis | N/A | 0.5;17 | 53.1 | Moderate |
| Akingbola TS | 2014 | Nigeria | Western | Cross-sectional | Prospective | No | Hospital-based | Urban | Both | Not mentioned | 20.5 | 11;30 | 44.9 | High |
| Diagne | 2001 | Senegal | Western | Cross-sectional | Retrospective | No | Hospital-based | Urban | Both | Not mentioned | N/A | N/A | N/A | High |
| de Montalembert | 1993 | Algeria | Northern | Cross-sectional | Retrospective | No | Hospital-based | Urban | Both | Hemoglobin electrophoresis | N/A | N/A | N/A | Moderate |
| Wahab K | 2012 | Nigeria | Western | Cross-sectional | Retrospective | No | hospital-based | urban | Both | Hemoglobin electrophoresis | 25 | N/A | 42.9 | Moderate |
| Ruffieux N | 2011 | Cameroon | Central | Cross-sectional | Prospective | No | hospital-based | urban | Both | Hemoglobin electrophoresis | 13.5 | 6;24 | 47.9 | Moderate |
| Njamnshi AK | 2006 | Cameroon | Central | Cross-sectional | Prospective | No | hospital-based | urban | Both | Hemoglobin electrophoresis | 13.49 | 0.6;35 | 54.17 | Low |
| Saidi H | 2016 | Tanzania | Eastern | Cross-sectional | Prospective | No | hospital-based | urban | Both | Hemoglobin electrophoresis | 6.36 | 1;14 | 58.1 | Moderate |
| Adamolekun B | 1993 | Nigeria | Western | Cross-sectional | Retrospective | No | hospital-based | urban | both | Hemoglobin electrophoresis | 11.2 | 2;45 | 50 | Moderate |
| Amayo EO | 1992 | Kenya | Eastern | Cross-sectional | Retrospective | No | hospital-based | urban | Both | Hemoglobin electrophoresis | 10.8 | 0.5;31 | N/A | Moderate |
| Djunga S | 1977 | DRC | Central | Cross-sectional | Retrospective | No | hospital-based | urban | both | Not mentioned | 7.6 | 0.15;18 | N/A | Hign |
| Izuora GI | 1989 | Nigeria | Western | Cross-sectional | Prospective | No | hospital-based | urban | both | Not mentioned | N/A |  | N/A |  |
| Adegoke SA | 2015 | Nigeria | Western | Cross-sectional | Prospective | No | hospital-based | urban | both | Not mentioned | 5.8 | 0,5;15 | 62.9 | Low |
| Fatunde OJ | 2005 | Nigeria | Western | Cross-sectional | Retrospective | No | hospital-based | urban | both | Hemoglobin electrophoresis | N/A | N/A | N/A | Moderate |
| Jude MA | 2014 | Nigeria | Western | Cross-sectional | Retrospective | No | hospital-based | Urban | both | Not mentioned | N/A | N/A | N/A | Moderate |
| Kehinde | 2008 | Nigeria | Western | Case-control | Prospective | No | Hospital-based | Urban | Both | Hemoglobin electrophoresis | N/A | N/A | 51.4 | Moderate |
| Rumaney | 2014 | Cameroon | Central | Case-control | Prospective | No | Hospital-based | Urban | Both | High performance liquid chromatography (HPLC) | 17.5 | N/A | 52.3 | Moderate |
| George | 2011 | Nigeria | Western | Cross-sectional | Retrospective | No | Hospital-based | Urban | Both | Hemoglobin electrophoresis | N/A | N/A | 58.2 | Moderate |
| Tantawy | 2015 | Egypt | Northern | Cross-sectional | Prospective | No | Hospital-based | Urban | Both | Hemoglobin electrophoresis | 11 | < 18 | 40 | Low |

N/A: not available or not applicable; Lagunju IA_1: published in Am J Hematol; Lagunju IA_2: published in Arch Dis Child; Lagunju IA_3: Int J Hematol
