# Supplementary material for: Neurologic complications of sickle cell disease in Africa: A systematic review and meta-analysis
Source: Neurology. 2017 Oct 3;89(14):1516–24. doi: 10.1212/WNL.0000000000004537 (PMC5631172; doi:10.1212/WNL.0000000000004537)
Supplement: Data Supplement [file supp_WNL.0000000000004537_Table_e-2.docx]

**Table e-2:** Studies reporting on the recurrence of stroke among sickle cell disease patients in Africa

| **Author, year of publication and country** | **Study design** | **Population** | **Prevalence of recurrent stroke among SCD patients with prior stroke** | **Prevalence of recurrent stroke among the general SCD population** | **Incidence of recurrent stroke among SCD patients with prior stroke** |
| --- | --- | --- | --- | --- | --- |
| Njamnshi et al, 2006, Cameroon | Cross-sectional | 120 SCD patients aged 7 months to 35 years; all HbSS | 25% (2/8) | 1.7% (2/120) | NA |
| Jude et al, 2014, Nigeria | Cross-sectional | 5,721 SCD patients | 23.9% (17/71) | 0.30% (17/5721) | NA |
| George et al, 2011, Nigeria | Cross-sectional | 256 SCD patients aged 6 months to 16 years | 18.2% (2/11) | 0.78% (2/256) | NA |
| Lagunju et al, 2012, Nigeria | Cross-sectional | 214 SCD children aged 15-199 months (median 105.5); 187 HbSS and 27 HbSC | 50% (9/18) | 4.2% (9/214) | NA |
| Lagunju et al, 2013, Nigeria | Prospective cohort | 31 SCD patients with previous stroke; 13 on hydroxyurea therapy (HU) with a mean duration of follow-up of 31.4 (SD=14.5) months and 18 not taking hydroxyurea (non-HU) with a mean duration of follow-up of 30.5 (SD=13.5) months | 77.8% (14/18) in the non-HU group and 15.4% (2/13) in the HU group | NA | 28/100 person-years in the non-HU group and 7/100 person-years in the HU group |

SCD: sickle cell disease; HU: hydroxyurea; NA: not available
