# Supplementary material for: Neurologic complications of sickle cell disease in Africa: A systematic review and meta-analysis
Source: Neurology. 2017 Oct 3;89(14):1516–24. doi: 10.1212/WNL.0000000000004537 (PMC5631172; doi:10.1212/WNL.0000000000004537)
Supplement: Accompanying Editorial [file supp_89_14_1516_v2_index.html]

Accompanying Editorial 

# Neurologic complications of sickle cell disease in Africa

## Accompanying Editorial

**Neurology® data supplements are not copyedited before publication. Published editorials and translations have been copyedited.  
 © 2017 American Academy of Neurology.  
  
 Files in this Data Supplement:**

- Accompanying Editorial - PDF
