# Supplementary material for: Neurologic complications of sickle cell disease in Africa: A systematic review and meta-analysis
Source: Neurology. 2017 Oct 3;89(14):1516–24. doi: 10.1212/WNL.0000000000004537 (PMC5631172; doi:10.1212/WNL.0000000000004537)
Supplement: Accompanying Editorial [file supp_WNL.0000000000004537_1439.pdf]

EDITORIAL

Sickle cell disease and the unmet challenges of neurologic complications

Gretchen L. Birbeck, MD
Ralph Green, MD, PhD

Correspondence to Dr. Birbeck: gretchen_birbeck@urmc.rochester.edu

Neurology® 2017;89:1439–1440

Sometimes an important scientific report is more notable for what it cannot tell us than for what it can. In this issue of *Neurology*®, Noubiap and colleagues[1] provide a comprehensive, well-conducted systematic meta-analysis of the neurologic complications of sickle cell disease (SCD) that incorporates rigorous design and advanced analytic methods to delineate in substantial detail what is known about the prevalence of neurologic injury among people with SCD in Africa—where over half the world's population of people with SCD reside. As one might expect, based upon what is known from more medically developed regions of the world, stroke, seizures, and headache are common. Data within the African meta-analysis offer some insights into why the stroke prevalence rates identified are more similar to populations from advanced medical settings than expected, given the lack of universal availability of the 2 best tried and proven modalities of both primary and secondary stroke prevention—adequate maintenance of low sickle cell hemoglobin levels through regular transfusion and hydroxyurea in resource-impoverished areas of Africa.

Within the African data in the meta-analysis, the temporal trends and prevalence rate comparisons across studies vary in terms of diagnostic sophistication and technological capacity, so that the 31 relatively high-quality studies identified likely underestimate neurologic complications. Places that have imaging available and that use more reliable case definitions have higher stroke prevalence. Epilepsy prevalence assessments appear to rise in conjunction with public awareness regarding epilepsy. These are gaps suggested within the available data—what do we not know?

In other regions, 39% of people with SCD have silent cerebral infarctions by 18 years of age. Recognition relies on neuroimaging, which remains largely unavailable in Africa outside capital cities and academic medical centers.[2] Although silent infarcts lack the immediate and overt motor phenomena, they affect executive function, intellect, and behavior.[3,4] Only 2 African studies to date address the cognitive consequences of SCD. All published data available come from hospital-based studies. The absence of population or even community-based data leaves unanswered the question of how the burden of SCD-associated neurologic sequelae might differ in such settings. One might surmise higher prevalence and severity of these sequelae with limited access to basic treatment (i.e., rural settings). Alternatively, hospital-based studies may sample from more severe disease. Given the dearth of neurologic expertise in much of Africa,[5] data from existing health information systems from more diverse clinical settings that include rural regions likely fail to capture the true prevalence of SCD neurologic sequelae. The design, implementation, and funding of studies adequately structured to ascertain SCD neurologic sequelae in Africa present major challenges. NIH funding allowed the establishment of the Sickle Pan-African Research Consortium in Tanzania, Ghana, and Nigeria, and represents an important first step.[6]

Several studies conducted in Western settings have characterized the neurologic consequences of SCD, especially stroke, but extrapolation of such data to the African population is not valid. The life course of Africans diverges widely from that of people in the United States because of varying factors, including environment, diet, and infectious exposures, to say nothing of health care provision for an underlying hematologic disorder. Furthermore, conflating Africa into a single region for scientific consideration, though often inevitable based upon existing information, is fraught with parochial hubris given the vast and heterogeneous nature of the continent. Importantly, unique risk factors and potentially novel environmental and even epigenetic protective factors for neurologic injury in SCD affecting either microbiota or gene expression may exist in Africa[7] and studies designed to identify these could provide critical insights into our understanding of this often devastating condition. New and exciting treatments of SCD, including hematopoietic stem cell transplantation, gene editing through zinc finger nucleases, and *CRISPR/cas9* gene editing are on the horizon.[8] Inevitably, these

See page 1516

From the Department of Neurology (G.L.B.), University of Rochester, NY; University Teaching Hospital Neurology Research Office (G.L.B.), University of Zambia, Lusaka; and Departments of Pathology and Internal Medicine (R.G.), University of California, Davis.

Go to Neurology.org for full disclosures. Funding information and disclosures deemed relevant by the authors, if any, are provided at the end of the editorial.

© 2017 American Academy of Neurology. Unauthorized reproduction of this article is prohibited.

options to ameliorate the global toll of SCD will serve, at first, to widen even further the chasm between those parts of the planet that have the resources to implement cutting-edge technologies and those that will need to make do with more affordable alternatives.

As is so often the case for neurologic reports from this part of the world, the take-home message is that better health outcomes will require improvements and investments in health systems, medical education, local scientific capacity, and public awareness. The neurologic status of individuals with SCD is predicated upon treatment, and the treatment gap for even basic care with hydroxyurea is not reported in this review and remains unknown. In 2008, the 63rd UN General Assembly discussed the burden of SCD in Africa and in 2009 an application for hydroxyurea to be placed on the WHO's Essential Medicines list was made,[9] but as of March 2017 neither hydroxyurea nor any SCD-specific therapies made the list.[10]

For all the limitations faced by Noubiap et al. in terms of the published data available for their analysis, this report draws attention to the visible neurologic consequences of SCD in Africa while also highlighting that there is an undoubtedly much larger burden. The neurology-savvy reader will recognize that this review details only the tip of the iceberg—or perhaps more appropriately, the ears of the hippopotamus.

STUDY FUNDING

No targeted funding reported.

DISCLOSURE

Drs. Birbeck and Green report no disclosures. Go to Neurology.org for full disclosures.

REFERENCES

1. Noubiap JJ, Mengnjo MK, Nicastro N, Kamtchum-Tatuene J. Neurologic complications of sickle cell disease in Africa: a systematic review and meta-analysis. Neurology 2017; 89:1516–1524.
2. WHO, IBE, ILAE. Atlas: Epilepsy Care in the World 2005. Geneva: World Health Organization; 2005.
3. Gold JI, Johnson CB, Treadwell MJ, Hans N, Vichinsky E. Detection and assessment of stroke in patients with sickle cell disease: neuropsychological functioning and magnetic resonance imaging. Pediatr Hematol Oncol 2008;25:409–421.
4. Quinn CT. Breakthrough: new guidance for silent cerebral ischemia and infarction in sickle cell disease. Hematol Am Soc Hematol Educ Program 2014;2014:438–443.
5. WHO/WFN. Atlas: Country Resources for Neurological Disorders. Geneva: WHO; 2004.
6. NIH_Reporter. U24HL135881. Bethesda, MD: NIH; 2017.
7. Kaddam L, FdleAlmula I, Eisawi OA, et al. Gum Arabic as fetal hemoglobin inducing agent in sickle cell anemia: in vivo study. BMC Hematol 2015;15:19.
8. Canver MC, Orkin SH. Customizing the genome as therapy for the beta-hemoglobinopathies. Blood 2016;127: 2536–2545.
9. Cheng Y, Walkom E. Proposal for the Inclusion of Hydroxyurea in the WHO Model List of Essential Medicines. Geneva: WHO; 2009.
10. WHO. WHO Model List of Essential Medicines, 20th List. Geneva: WHO; 2017.

© 2017 American Academy of Neurology. Unauthorized reproduction of this article is prohibited.
